# Adolescent health literacy: factors effecting usage and expertise of digital health literacy among universities students in Pakistan

**DOI:** 10.1186/s12889-020-10075-y

**Published:** 2021-01-09

**Authors:** Adnan Adil, Ahmed Usman, Nasir Mehmood Khan, Faria Ibad Mirza

**Affiliations:** 1 Institute of Social & Cultural Studies, University of the Punjab, Lahore, Pakistan; 2 Sociology, Institute of Social & Cultural Studies, University of the Punjab, Lahore, Pakistan; 3 PMAS Arid Agriculture University, Rawalpindi, Pakistan; 4 Lecture Sociology, PMAS Arid Agriculture University, Rawalpindi, Pakistan

**Keywords:** eHealth, Digital health literacy, University students, Usage of electronic health

## Abstract

**Background:**

Electronic health tools are of little use if the intended user lacks the skills to effectively engage them. Engaging eHealth requires a skill set, or literacy, of its own. The present study is an effort to probe the relationship of education and Institution (Independent Variables) with the usage and expertise in eHealth literacy (Dependent Variables) among university students. The research is conducted in 16 Higher Education Commission (HEC) Pakistan’s recognized universities in Lahore. Both male and female students ranging from BS to PhD programs were the focus of the research.

**Methods:**

Quantitative data was collected through survey method using stratified random sampling technique. There were different kinds of strata in population i.e. general universities, health sciences universities, engineering universities and animal sciences universities etc. The research encompassed a total of 89,664 students in 16 universities, from which sample size of 1513 was drawn through research advisor table (2006). Proportional allocation formula was used to specify the number of respondents from each university. Non-parametric statistics was used since data was not normally distributed. Kruskal-Wallis H test and Mann-Whitney U test were applied to measure the difference of effect of groups of independent variables on the dependent variables.

**Results:**

The level of using digital health literacy was not same for all students, as the students of PhD and BS/Masters were significantly different from each other in terms of their usage of digital health literacy. Level of education showed a significant influence on level of expertise in eHealth literacy, confirming that changing the level of education had an effect on level of expertise in digital health literacy, but the size of effect was smaller. MS/MPhil and PhD students were significantly different from each other in their expertise in digital health literacy.

**Conclusion:**

Results of the study depicted that belonging to different categories of educational levels differently affect the level of usage and that of expertise in digital health literacy among university students.

## Background

Digital health literacy (DHL) denotes the level to which individuals have the capability to contact, rehearsal, and grasp the elementary health knowledge, and amenities essential to make apt health judgments while using electronic resources [1]. DHL is developing as a significant concept that can upshot into outstanding optimistic vicissitudes in health. It was espoused by industrialized nations first, and nowadays the emerging nations are also making efforts to fit-in it into their health system [2]. According to the reports of ‘Pew Internet and American Life Project’, the world population is increasing at the pace of 1.4% per annum, while technology users are growing at the pace of 7.9% per annum. This gap depicts that inclination towards adoption of advanced technologies is becoming an ideal source of getting knowledge [3].

The practice of using android cell phones and other such components had also paved the ways to get online health information. According to the statistics of ‘Pew Internet’, 52% percent smart android mobile users had surfed for online health knowledge through their mobiles for more than one time. Other statistics of ‘Pew Internet’ further illustrate that 10 out of 100 persons are consuming internet, and out of these 10 personages, seven individuals falls under the definition of youth [3], whereas as per the statistics of Ministry of Youth Affairs, Pakistan (2017) [4], approximately one third part of population belongs to youth segment. Findings from the previous studies advocates that apprentices, scholars, and patients etc. can be characterized as the recipients of the digital health knowledge [5]. Practice of these ehealth properties is greatly influenced by gender [6], internet access, level of education, and technical support in usage of e-resources [7]. Dickerson et al., [8] depicted the association of level of education and ethnicity of patients, with online health literacy. The patients were divided into two diverse groups i.e. who attended college, and who didn’t. They made conclusion that the patients, who had appeared in college, had a better level of practice in digital health literacy, whereas the patients, who had not attended college in their life, had lower level of usage of online health literacy. The study of Fogel et al., [9] came-up with the same findings, depicting the association of education and income, with DHL. Similarly, the logitudinal study of Flynn et al., [10] pointed out a positive association between higher level of education and online health literacy.

The contextual understanding of the literature depicted that ICTs usage was associated with level of education. The present study intended to probe the factors associated with ICTs usage in searching health. The present study hypothesized that the level of education is associated with (1) level of usage and (2) level of expertise in digital health literacy among university students in Pakistan.

## Methods

For the present study, there is a single categorical independent variable (level of education) having three categories i.e. BS/Masters, MPhil and PhD, while there are two continuous dependent variables i.e. level of usage and level of expertise in digital health literacy. Level of usage of digital health literacy was measured with a scale of 6 items, using a four point Likert scale from 1 = “very often” to 4 = “never”, and level of expertise in digital health literacy was measured with a scale of 6 items, using a four point Likert scale from 1 = “strongly disagree” to 4 = “strongly agree”.

The study was conducted in Lahore, Pakistan, as there are number of public and private sector universities located in the city. According to HEC list 2013, there are 16 public and private sector universities in Lahore [11]. There were four different types of universities in Lahore i.e. general universities, health sciences universities, engineering universities and animal sciences universities.

Stratified random sampling was employed for the selection of respondents as the categories in our population were heterogeneous. The sub population within an overall population varied. The total number of students in 16 universities was 89,664 (As depicted in Table 1). Sample size (*n* = 1513) was calculated through Research Advisor Table 2006 [12], while keeping 95% confidence interval and 2.5% margin of error. Formula of proportionate allocation determined the number of respondents from each stratum.

$$ ni=n\left(\frac{Ni}{N}\right) $$

**Table 1:** University wise allocation of sample

Strata	Universities Name	Students in Selected University	Selected Students in Sample
	University of South Asia, Lahore	1171	20
	University of Management and Technology, Lahore	3308	56
	University of Lahore, Lahore	11,461	193
	University of Central Punjab, Lahore	4237	71
	Minhaj University, Lahore	1464	25
	Lahore University of Management Sciences	2356	40
	Hajvery University, Lahore	2758	47
	Beaconhouse National University, Lahore	1178	20
	University of Education, Lahore.	10,369	174
	University of the Punjab, Lahore	24,961	421
	Government College University, Lahore	7438	125
	Lahore College for Women University	4582	**77**
Health sciences	King Edward Medical University	1826	31
	University of Health Sciences, Lahore	156	4
Engineering	University of Engineering and Technology	9399	158
Animal Sciences	University of Veterinary and Animal Sciences	3000	51
Total	89,664		**1513**

Where:

*ni*= sample size for each stratum, *n* = sample size, *Ni* = Stratum Size, *N* = Population Size.

Respondents completed a self-report questionnaire [13] containing scales measuring expertise and usage level of digital health literacy. Respondents were also asked questions regarding their educational level, time spent on computer and type of institution. Face validity and factor analysis were used to measure the validity of data collection tool. Face validity means to what extent it looks like to measure the desired thing [14]. For this purpose, many teachers including PhD supervisor, were consulted. To measure the validity, factor analysis was also performed. While measuring the components of data collection tools, approximately all the components fell in the level of I component extraction, which declared the validity of data collection tool. Then value of Alpha (value of **α** > 0.7) declared the reliability of the instrument. The value of alpha (α) was obtained through checking cross-product deviations and covariance in SPSS.

## Results

Statistics of Table 2 are depicting that total number of the respondents was 1513. Majority of students i.e. 1269 (83.9%) belonged to general universities, while 51 (3.4%) were from animal sciences universities, 158 (10.4%) from engineering universities, and 35 (2.3%) respondents were from health sciences universities. Similarly, 730 (48.2%) students were in their BS/Masters degree, 679 (44.9%) in MS/MPhil degree and 104 (6.9%) in PhD degree.

**Table 2:** Descriptive Analysis for Socio-Cultural Variables

Socio-Cultural Characteristics	Frequency	Percentage
**Type of Institution**		
Animal Sciences Universities	51	3.4
General	1269	83.9
Engineering	158	10.4
Health Sciences	35	2.3
Total	1513	100
**Education**		
Bs/Masters	730	48.2
Ms./Mphil	679	44.9
PhD	104	6.9
Total	1513	100.0
**Time Spent on Computer**		
0–1	28	1.9
2–3	468	30.9
4–5	658	43.5
6=>	359	23.7
Total	1513	100.0

Furthermore, the data is also elaborating that 28 (1.9%) respondents were spending 0 to 1 h daily on computer, while 468 (30.9%) respondents were spending 2 to 3 h daily on computer. A significant number of respondents 658 (43.5%) was spending 4 to 5 h daily on computer, while 359 (23.7) percent respondents were spending 6 or more hours on computer daily. Data is showing that approximately 75% of the respondents were spending 2 to 5 h daily on computers.

### Statistical analysis for hypothesis 1

Data in Table 3 is depicting the association between level of education and level of usage of digital health literacy. The independent variable was categorical having three different educational groups i.e. M.A, MPhil., and PhD, while dependent variable was continuous. The purpose was to observe whether changing the level of education had an effect on the level of usage of digital health literacy among students or not? The statistics Kruskal-Wallis H test depicted a gap in the means rank (621 to 771), which strengthened the argument that means of different levels of education were different for level of using digital health literacy. P. value (.004) rejected the null hypothesis and concluded that the students belonging to different classes had different level of usage in digital health literacy.

**Table 3:** Relationship between Level of Education and Level of Usage of Digital Health Literacy

	Education	Mean Rank
**Level of Usage**	BS/Masters	763.09
	MS/M.Phil.	771.22
	PhD	621.37

Test Statistics: Chi- Square: 11.003, df: 2, P. value: .004

The value of Dunn test value (0.28) (chi-square value (11.003) was divided by $\sqrt{n}$ (Under root sample size) depicted a medium size of effect by independent variable on the dependent variable.

Data in Table 4 is depicting the test statistics of Mann-Whitney U test. The data showed that students of PhD and BS/Masters were significantly different from each other in the usage of digital health literacy. Similarly, PhD students were also significantly different from MS/MPhil students in level of usage of digital health literacy. Such findings concluded that level of usage of digital health literacy was different among students of different classes.

**Table 4:** Mann-Whitney U Tests for Level of Education and Level of Usage of Digital Health Literacy

	Level of Education	***P***-Value
**Level of Usage**	BS / Master – MS / M.Phil.	.695
	BS / Masters – PhD	.001
	MS / M.Phil.	.002

### Statistical analysis for hypothesis 2

Data in Table 5 is depicting the effect of categorical independent variable (level of education) on the continuous dependent variable (level of expertise in digital health literacy). The aim was to examine whether changing the level of education had an effect on level of expertise in digital health literacy or not? Kruskal-Wallis H. test statistics depicted a gap in the mean ranks (682 to 782), authenticating that changing the level of education had an effect on level of expertise in digital health literacy. P. Value (.044) also rejected the null hypothesis.

**Table 5:** Relationship between Level of Education and Level of Expertise in Digital Health Literacy

	Education	Mean Rank
**Level of Expertise**	BS/Masters	743.48
	MS/M.Phil.	782.98
	PhD	682.26

Test Statistics: Chi- Square: 6.250, df: 2, P. value: .044

Dunn test value (0.1) (Chi-square value (6.250) was divided by $\sqrt{n}$) depicted a smaller size of effect of independent variable on the dependent variable. It was estimated that the level of expertise was affected by the level of education, but at a smaller level. To analyze, which groups of independent variable were affecting the dependent variable differently, Mann-Whitney U test was applied. For this purpose, groups of Independent variables were categorized pair-wise. Then each pair’s effect was analyzed against the dependent variable.

Data in Table 6 shows that MS/MPhil and PhD students were significantly different from each other in their level of expertise in digital health literacy that authenticated the argument that students belonging to different levels of education were different in level of expertise in digital health literacy.

**Table 6:** Mann-Whitney U Test for Level of Education and Level of Expertise in Digital Health Literacy

	Level of Education	P-Value
**Level of Expertise**	BS/ Masters – MS / MPhil	.088
	BS / Masters – PhD	.180
	MS / MPhil – PhD	.026

## Discussion

In contrast to previous researches, the present research included a larger sample in order to examine the factors effecting usage and expertise of digital health literacy among students. In addition to finding the relationship between independent and dependent variable, the present study investigated the relative difference within the categories of independent variable during their effect on the dependent variable.

In line with previous researches, the findings of the present study found that the level of usage of digital health literacy was different among the students of different educational categories. Students of PhD and BS/Masters were significantly different from each other in terms of their usage of digital health literacy. In the same manner, PhD students were also significantly different from MS/MPhil students in terms of their level of usage of digital health literacy. Previous studies suggested similar results. The study of Wangberg et al., [7] investigated the relationship between education and subjective health information; education was found to be more closely related to subjective health information. Similarly, Matsuyama et al., [15] pointed out the affiliation of education of patients with digital health literacy. They concluded that the patients who attended college in their life had higher use of technology for accessing health information as compared to those who did not attend the college. Similarly, Fogel et al., [9] examined the occurrence and predictors of internet use for medical information among women with breast cancer. They found that patients with higher education or income, and patients of white race were more likely to use the internet for breast cancer related health issues. Flynn et al., [10] tried to understand how and when patients use non-physician sources of health. It was concluded that years of education were associated with searching online for health information. Similarly, James et al., [16] examined cancer patients’ and carers’ use of, and attitudes towards, the internet as a health information source. The use of internet information was uniformly low among ethnic minorities and was uniformly high among educated patients.

The present research complements previous researches by highlighting the association between level of education and level of expertise in digital health literacy. It is found that MS/MPhil and PhD students are substantially dissimilar to each other in their level of expertise in digital health literacy. Similar results were drawn by Birru et al., [17]; they concluded that low literacy may come across as an informational hurdle on the internet when searching for health information because most health related web sites necessitate at least a high-school reading proficiency for best possible access. Similarly, Gustafson et al., [18] reviewed different reports of comprehensive Health Enhancement Support System (CHESS) and concluded that the higher level of education was required to benefit from digital health literacy. Once again, same kind of unexpected results emerged as increasing or decreasing the level of education did not increase or decrease the level of expertise in digital health literacy. However, it was found through values of mean ranks that the usage and the expertise level in digital health literacy are positively associated.

The limitations of the present study propose guidelines for future research. It would be interesting to extend this study to other contexts or institutions of education. It is also important to extend the research by including other related variables such as students’ age, gender, and ICT expertise etc. The current study could only collect data from the university students of different disciplines and institutions. The future studies should focus to produce results in a larger scenario including school and college students. It is furthermore suggested that the validity of the present study can be enhanced by taking the opinion of students having some kind of permanent disease.

## Conclusion

This study found that there is an association between the level of education and the levels of (1) usage and (2) expertise in digital health literacy. Students of diverse educational levels had diverse levels of usage and expertise in the digital health literacy. On the basis of findings, the study concludes that educational level is the major factor for unequal response towards digital health literacy. The study furthermore depicted that the students of BS/Master, MS/MPhil and PhD are substantially different from each other in their level of usage and expertise in digital health literacy. The findings of the present research can play a vital role in narrowing health inequalities. The study may also provide guidelines to health policy makers by indicating that the educational level can promote general health searching tendencies among the general masses.

## Data Availability

The datasets used during the current study are available from the corresponding author on reasonable request.

## Abbreviations

DV: Dependent Variable
DHL: Digital health literacy
HEC: Higher Education Commission
IV: Independent Variable
ICTs: Information and Communication Technologies
